# ENKUR acts as a tumor suppressor in lung adenocarcinoma cells through PI3K/Akt and MAPK/ERK signaling pathways

**DOI:** 10.7150/jca.30021

**Published:** 2019-07-05

**Authors:** Qing Ma, Yin Lu, Jie Lin, Ye Gu

**Affiliations:** 1 College of Biological and Environmental Engineering, Zhejiang Shuren University, Hangzhou, Zhejiang 310015, P.R. China; 2 Department of Gastroenterology, Affiliated Hangzhou First People's Hospital, Zhejiang University School of Medicine, Hangzhou, Zhejiang 310006, P.R. China.

**Keywords:** Cellular behavior, EMT, ENKUR, lung adenocarcinoma, tumorigenesis

## Abstract

Lung adenocarcinoma (LAD) is the most common type of lung cancer with a high possibility of tumor relapse and metastasis. ENKUR (Enkurin) was originally identified as a potential regulator or effector of TRPC channels that directly binds to several TRPC proteins and the p85 subunit of PI3K. However, the role of ENKUR in cancer development has remained unclear. In this study we analyzed the expression profile of *ENKUR* mRNA in clinical LAD samples and examined *ENKUR* mRNA expression and ENKUR protein level in LAD cells. Significant down-regulated *ENKUR* expression was observed in clinical tumor tissues of LAD as well as in human LAD cells. To evaluate the effects of aberrant ENKUR expression on cellular biology of LAD cells *in vitro* and tumor growth *in vivo*, ENKUR-overexpressed and -silenced LAD cell lines were constructed using lentiviral vectors. Our results showed that overexpression of ENKUR in LAD cells inhibited cell proliferation, migration and invasion, while silencing of ENKUR led to the opposite effects. Silencing of ENKUR in LAD cells also promoted tumorigenesis in nude mice model and caused epithelial to mesenchymal transition (EMT). Furthermore, using western blot and co-immunoprecipitation analyses, we demonstrated that ENKUR interacts with PI3K directly and is possibly involved in the PI3K/Akt and MAPK/ERK signaling pathways.

## Introduction

In the past decades, lung cancer remains the leading cause of cancer-related mortality worldwide 1. Lung adenocarcinoma (LAD) is the most common type of lung cancer 2. A recent study has showed that the occurrence of LAD is stabilizing in men but still increasing annually in women 3. Due to the high possibilities of tumor invasion and migration, patients with LAD often die from relapse and metastasis 4. Although development of chemotherapy, radiotherapy, and targeted therapy combined with surgery have shown benefits for patient overall survival, the likelihood of a complete cure is slim 5, 6. The development of effective diagnostic markers and novel therapeutic approaches relies on a thorough understanding of the molecular mechanisms underlying LAD initiation and progression.

The *ENKUR* (*Enkurin*) gene is located at chromosomal band 10p12.1 and is firstly identified by yeast two-hybrid screening in a study of transient receptor potential canonical (TRPC) 7. ENKUR protein interacts with several TRPC proteins and acts as a potential regulator or effector of TRPC channels. Three potential protein-protein interaction sites in ENKUR were identified from the deduced amino acid sequences and confirmed by GST pull-down assay. These three interaction domains include a C-terminal region involved in channel interaction, an IQ motif related with the Ca^2+^ sensor, and a proline-rich N-terminal region that allows ENKUR to directly interact with the p85 regulatory subunit of Phosphoinositide 3-kinase (PI3K) 7. ENKUR was thus hypothesized as an adapter protein linking signal transduction proteins with TRPC channels 7. However, so far, the function and mechanism of *ENKUR* in human cancer has remained unclear.

In this study, we analyzed the expression profile of *ENKUR* mRNA in clinical LAD samples from The Cancer Genome Atlas (TCGA) database and the Oncomine platform and examined *ENKUR* mRNA expression and ENKUR protein level in human LAD cells. We also investigated the effects of aberrant ENKUR expression on the biological behavior and tumor-forming ability of LAD cells and revealed potential involvement of ENKUR in the MAPK/ERK and PI3K/Akt signaling pathways.

## Methods

### Ethics statement

The study was carried out strictly according to National Institutes of Health (NIH) Guidelines for Laboratory Animal Care 8 and Guidelines for the welfare and use of animals in cancer research 9. The protocol was approved by the Ethic Committee of Zhejiang Shuren University. All efforts were made to minimize animal suffering and to reduce the number of animals used.

### UALCAN analyses based on TCGA dataset

The web-portal UALCAN (http://ualcan.path.uab.edu/index.html) was accessed to obtain in-depth analyses of *ENKUR* mRNA expression profiles from the TCGA database (cancergenome.nih.gov) 10. The expression level of *ENKUR* mRNA was examined in various types of clinical tumor samples, including lung adenocarcinoma (LAD), lung squamous cell carcinoma, colon adenocarcinoma, kidney chromophobe, and thyroid carcinoma. The expression profile of LAD includes 515 tumor tissue samples and 59 non-tumor lung tissue samples. All the clinical information of enrolled patients for expression analyses, including age, gender, histology, pathological stage as well as the corresponding biospecimen are available in the TCGA Data Portal with the project ID: TCGA-LUAD.

### Oncomine data analysis

The expression level of *ENKUR* in LAD was also analyzed using Oncomine (https://www.oncomine.org/resource/login.html), a web-based data-mining platform aimed at facilitating discovery from genome-wide expression analyses 11. Clinical cases of LAD in the Oncomine database with statistical significance (*p*<0.05) were enrolled in this study. Datasets with significant expression differences (*p*<0.05) between clinical LAD samples and the paired normal lung tissues were selected for further analysis to evaluate the correlation between *ENKUR* expression and pathological stage.

### Cell culture

Two human LAD cell line A549 and H322 were obtained from the American Type Culture Collection (Manassas, VA, USA) and the European Collection of Authenticated Cell Cultures (Porton Down, Salisbury, UK), respectively. One human LAD cell line PC9 and one normal human bronchial epithelial cell line 16HBE were obtained from the Institute of Biochemistry and Cell Biology (Shanghai, China). Two human LAD cell lines SPC-A1 and GLC-82 were obtained from the National Infrastructure of Cell Line Resource (http://www.cellresource.cn; Beijing, China). All cell lines used in this study were authenticated using short tandem repeat (STR) profiling provided by the cell bank. Cells were used for all experiments soon after receipt or resuscitation within 6 months. The RPMI 1640 medium containing 10% heat-inactivated fetal bovine serum (FBS) was used for cell culture in a humidified incubator at 37^o^C with 5% CO_2_.

### Quantitative real-time PCR (qRT-PCR)

Total RNA was extracted from cells using the Trizol® reagent (Invitrogen, Carlsbad, CA, USA) following the manufacturer's instructions. The extracted total RNA was reverse transcribed using the RevertAidTM First Strand cDNA Synthesis Kit (Fermentas, Ontario, Canada). Real-time quantitative PCR (qRT-PCR) was performed in an ABI StepOne Plus Real-Time PCR System (Applied Biosystems, Foster City, CA, USA) using the Platinum SYBR green master mix (Invitrogen) according to the manufacturer's instructions. Each reaction mixture contains 10 μl of Supermix, 0.8 μM of each primer and 0.1-0.5 μg of template cDNA. The primer sequences are provided in Table 1. The amplification procedure includes an initial denaturation step for 2 min at 95ºC, followed by 40 cycles of denaturation for 30 sec at 95ºC, annealing for 45 sec at 55ºC, and extension for 30 sec at 72ºC, with a final extension stage for 10 min at 72ºC. Relative mRNA expression was calculated using the ΔΔCt method with *β-ACTIN* as internal control 12. All tested samples were technically triplicated, and each experiment was conducted with three biological replicates.

### Western blot analyses

Total protein was extracted from approximately 1×10^7^ cells after rinsing with ice-cold phosphate-buffered saline. Cells were lysed on ice in radio immunoprecipitation assay (RIPA) buffer and protein concentrations were determined using the Bradford reagent (BioRad, Hercules, CA, USA). Protein lysates were resolved on 12% SDS polyacrylamide gels and electro-transferred to polyvinylidene fluoride membranes (ImmobilonP; Millipore, Bedford, MA, USA). After treatment with 5% non-fat dry milk in Tris-buffered saline, the membranes were immunoblotted with the following antibodies: PI3 Kinase p85 (1:1000, #4292, Cell Signaling, Danvers, MA, USA); Akt (1: 1000, #9272, Cell Signaling, Danvers, MA, USA); pAkt (1: 2000, #4060, Cell Signaling, Danvers, MA, USA); E-cadherin (1:500, #SAB4503751; Sigma Aldrich, St. Louis, MO, USA); N-cadherin (1:5000, # SAB2702400; Sigma Aldrich, St. Louis, MO, USA); vimentin (1:200, #V6630; Sigma Aldrich, St. Louis, MO, USA); ERK1/2(1: 1000, #sc-16981-R, Santa Cruz Biotechnology, Santa Cruz, CA, USA); pERK1/2 (1: 1000, #sc-7383, Santa Cruz Biotechnology, Santa Cruz, CA, USA); ENKUR (1:200, #HPA161503; Sigma Aldrich, St. Louis, MO, USA); β-ACTIN (1: 5000, #A5441, Sigma Aldrich, St. Louis, MO, USA). Then the membranes were incubated with the appropriate secondary antibodies (HRP-conjugated anti-rabbit IgG, 1:2000, #7074; HRP-conjugated anti-mouse IgG, 1:2000, #7076; Cell Signaling, Danvers, MA, USA). The blots were finally visualized by enhanced chemiluminescence method (Pierce, Rockford, IL, USA) following the manufacturer's instructions.

### Co-immunoprecipitation

Cell lysates were prepared as described above from LAD cell line A549. The cell lysates were pre-cleared by incubating with pre-blocked Protein A Sepharose beads (Zymed). Then, individual antibodies (PI3K, 1:1000, #4292, Cell Signaling; ENKUR, 1:200, #HPA161503, Sigma Aldrich; normal rabbit IgG, #2729, Cell Signaling) were added and incubated overnight at 4°C before harvesting of complexes with protein A Sepharose (GE Healthcare) and brief centrifugation. Bound proteins were separated with SDS/PAGE, followed by visualization using western blotting.

### Lentiviral transduction and transfection

The pEZ-Lv105 and psi-LVRH1GP shRNA lentiviral vectors (GeneCopoeia, Carlsbad, CA, USA) were used to construct ENKUR-overexpression and -silenced cell lines with HEK293T as the packaging cell line. The shRNA sequence is as follows: CCGGCCAACCTCGATACTCTTATTTCTCGAGAAATAAGAGTATCGAGGTTGGTTTTTTG. Recombinant lentiviruses were produced by transient transfection of HEK293T cells with the lentiviral vector by calcium phosphate method. Transduced cells were selected in medium containing 1 µg/ml puromycin. Quantitative real-time PCR and western blot analyses were performed to test the overexpression and silencing efficiencies.

### Cell proliferation assay

Cells were seeded at a density of 1 × 10^3^ cells per well containing 100 μl of culture medium in 96-well plates. The plates were shaken on a microplate shaker for 1 to 7 days at 37^o^C in a humidified atmosphere with 5% CO_2_. On each day, 20 μl of 5 g/l 3-(4, 5-dimethylthiazol-z-yl)-2, 5-diphenyltetrazolium bromide (MTT) (Sigma Aldrich, St. Louis, MO, USA) was added into each well. The plate was incubated for a further 4 h before removal of MTT and extraction of formazan into 150 μl of dimethyl sulphoxide (DMSO) (Sigma Aldrich, St. Louis, MO, USA). The number of cells at each time point was assessed by measuring the absorbance of the wells at 450 nm using a Microplate Autoreader (Bio-Rad, Hercules, CA, USA). The experiment was performed in triplicates.

### Colony formation assay

Cells were seeded at a density of 2 × 10^2^ on six-well culture plates and incubated at 37^o^C for 2 weeks. Then the cells were washed twice with PBS and fixed and stained with haematoxylin. The number of colonies containing ≥ 50 cells was counted using a microscope. The experiment was performed in triplicates.

### Cell wound healing assay

Cells were plated on six-well tissue plates (1.25 × 10^6^ cells per well) and cultured for 24 h until grown to 80 - 90% confluence. Then a uniform scratch or a “wound” was created across each well using a 10 μl standard pipette tip. The detached cells were removed, and the cells were allowed to grow for another 24 h. During this period, wound margins were photographed, and cell migration was monitored using an inverted microscope. Images were captured using an image-analyzing frame-grabber card (LG-3 Scientific Frame Grabber; Scion, Frederick, MD, USA). The unfilled scratched zones were quantified by measuring the distance between the advancing margins of cells using the image analysis software NIH Image 1.55. The scratch was photographed in three randomly selected microscopic fields (×200) at each time point. The experiment was performed in triplicates.

### Transwell migration assay

About 5 × 104 cells were suspended in serum-free medium and plated in the upper chamber of 8-μm-pore Transwells (Costar, Corning, Cambridge, MA, USA). 300 μl of 10% FBS in free medium (Gibco, Invitrogen, Carlsbad, CA, USA) was added to the lower chamber. To determine the amount of invasion, cells were incubated for 24 h and then removed from the upper chamber using a cotton swab. The invaded cells in the lower chamber were fixed with 1% paraformaldehyde and stained with haematoxylin. The downsides of the membrane were then photographed, and the number of transmigrated cells was counted in five randomly selected fields (×200) under a microscope. The experiment was performed in triplicates.

### Xenograft growth assay

To investigate whether ENKUR affects tumor growth *in vivo*, 1×10^6^ cells from the ENKUR-silenced LAD cell line A549-shRNA together with its control group were implanted into 4-week-old nude mice through subcutaneous injection. Twelve nude mice kept under routine laboratory condition (21-22^o^C, relative humidity 60% and a 12 h light-dark cycle) without disability, infection or inflammation were randomly allocated into the experimental and the control group. The health condition of the mice and primary tumor growth were monitored on each day. The resultant tumor size was measured from two perpendicular axes using a caliper and the tumor volume was calculated using the formula volume=^1^/_2_ (length × width^2^. All mice were euthanized using CO_2_ inhalation according to animal care guidelines.

### Statistical analysis

Statistical analyses were conducted using SPSS version 20 (SPSS Inc, Chicago, IL, USA). Student's *t*-tests were used to compare the expression levels of ENKUR and cell proliferative, invasive, and migratory rates between two different groups. A threshold value was set at 0.05 (two-tailed).

## Results

### *ENKUR* expression is down-regulated in clinical samples of LAD and other cancer types

**Figure 1:**
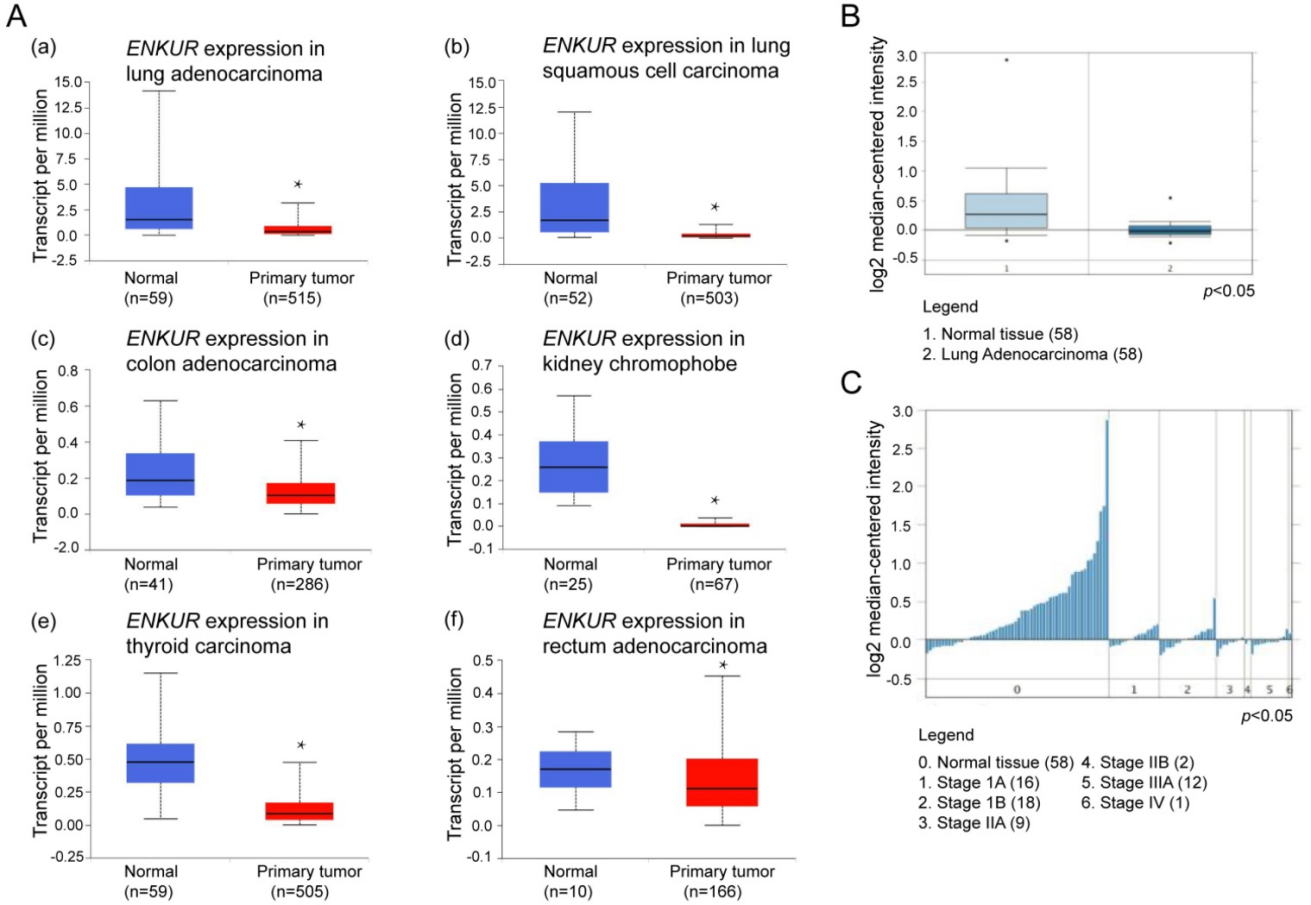
** UALCAN and Oncomine analyses of *ENKUR* in LAD.** (A) Expression profiling of *ENKUR* in different cancer types from UALCAN based on the TCGA database. *ENKUR* expression was significantly down-regulated in (a) lung adenocarcinoma (LAD); (b) lung squamous cell carcinoma; (c) colon adenocarcinoma; (d) kidney chromophobe; (e) thyroid carcinoma; (f) rectum adenocarcinoma. (B) and (C) Expression profiling of *ENKUR* in the Oncomine database. All the clinical cases demonstrated statistically significant differences in the expression level of *ENKUR* between tumor tissues and normal tissues with a *p*-value less than 0.05. (B) *ENKUR* expression was significantly lower in 58 cases of clinical LAD samples than in the paired normal tissues. (C) The expression level of *ENKUR* in LAD was negatively correlated with pathological stage. * *p* < 0.05.

Expression of *ENKUR* in various types of clinical tumor tissues were analyzed using the UALCAN portal, which is a newly developed interactive web server for analyzing RNA sequencing data in the TCGA database. Compared with normal non-tumor lung tissues, *ENKUR* showed significant down-regulation in tumor tissues of lung adenocarcinoma (LAD), lung squamous cell carcinoma, colon adenocarcinoma, kidney chromophobe, and thyroid carcinoma (*p* < 0.05; Figure 1A).

Next, we explored the expression of *ENKUR* mRNA in human tumor tissues using Oncomine, a cancer microarray database, which provides access to a large collection of cancer profiling datasets and analytical tools. Significant down-regulation of *ENKUR* was reported across 60 datasets in lung cancer, breast cancer, cervical cancer, colorectal cancer, pancreatic cancer, brain cancer, head and neck cancer, lymphoma, melanoma, and myloma (*p* < 0.05). Among these different cancer types, an expression profile of LAD including 58 clinical tumor tissue samples and the paired normal tissues was further examined 13. *ENKUR* showed significantly lower expression in clinical LAD tissues (*p* < 0.05; Figure 1B). Meanwhile, the expression level of *ENKUR* was lower in tumor tissues with higher pathological stage (*p* < 0.05; Figure 1C).

### ENKUR expression is down-regulated in LAD cells

**Figure 2:**
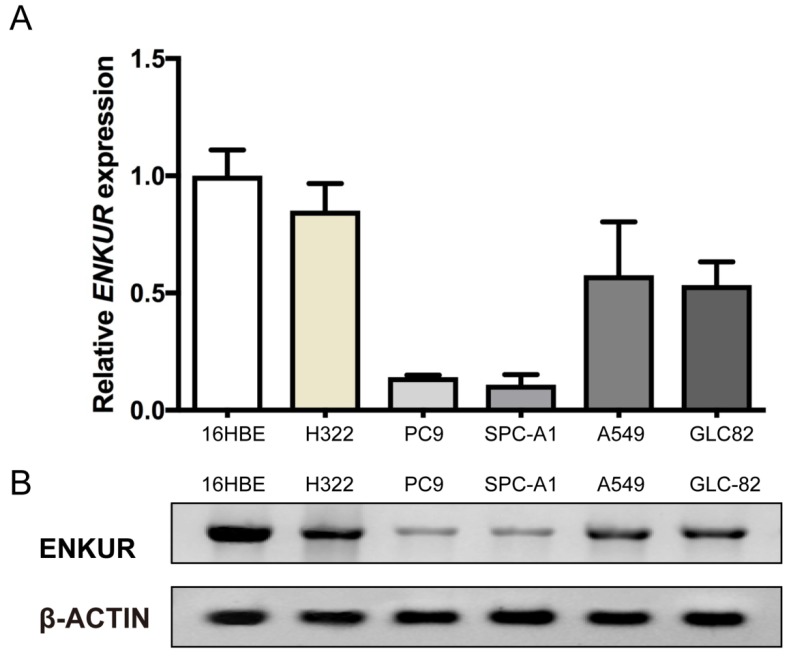
***ENKUR* mRNA expression and ENKUR protein level in five LAD cell lines and one human bronchial epithelial cell line.** (A) qRT-PCR analysis of* ENKUR* expression at the mRNA level. (B) Western blot analysis of ENKUR expression at the protein level. Each bar represents the mean ± S.D. of three replicates.

Western blot and qRT-PCR analyses were performed to examine the expression levels of ENKUR in five LAD cell lines, A549, PC9, H322, SPC-A1, and GLC-82, as well as the human bronchial epithelial cell line 16HBE. ENKUR expression was down-regulated in all the five LAD cell lines as compared with the normal cell line. Among the five LAD cell lines, ENKUR expression was the highest in H322 and the lowest in PC9 cells (Figure 2).

### Overexpression of ENKUR represses the proliferation, migration, and invasion of human LAD cells

**Figure 3:**
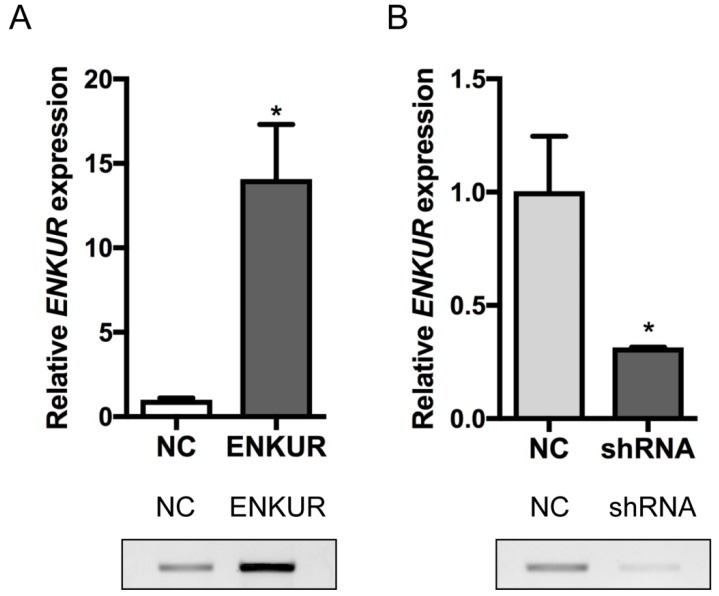
** Overexpression and silencing efficiencies of ENKUR in A549 cells.** (A) *ENKUR* mRNA expression and ENKUR protein level in ENKUR-overexpressed A549 cells (ENKUR) and control cells (NC) assessed by qRT-PCR and western blot analyses. (B) *ENKUR* mRNA expression and ENKUR protein level in ENKUR-silenced A549 cells (shRNA) and control cells (NC). Each bar represents the mean ± S.D. of three replicates. * *p* < 0.05.

**Figure 4:**
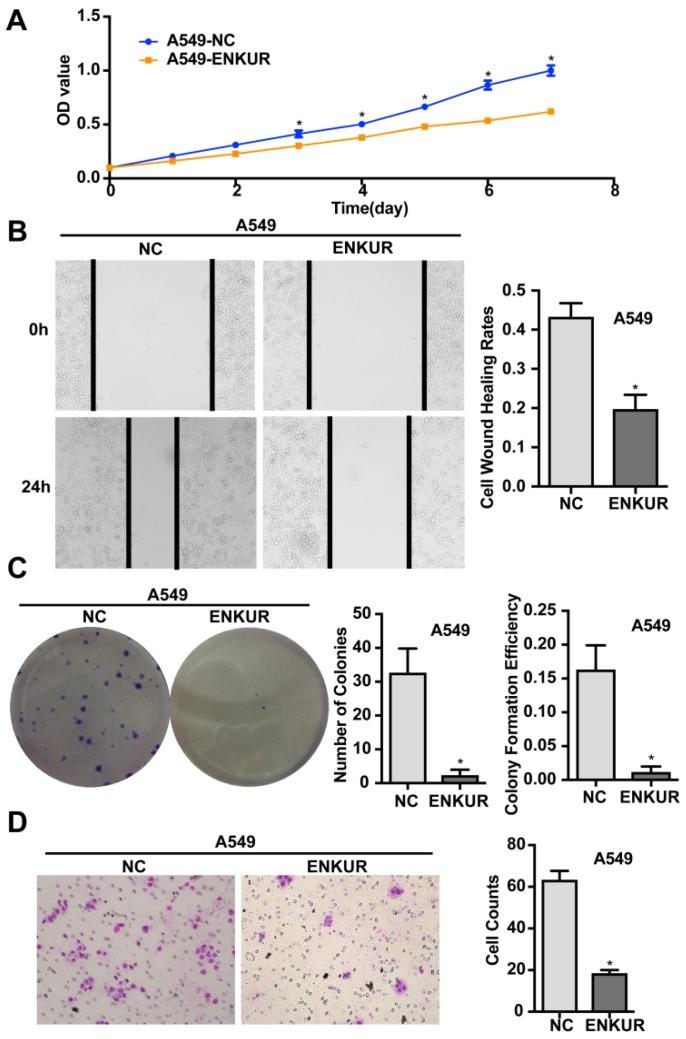
** Overexpression of ENKUR inhibits proliferation, migration, and invasion of LAD cells.** (A) ENKUR-overexpression cells A549-ENKUR exhibit decreased cell proliferation compared with the control groups (NC) as determined by the MTT assay. (B) Overexpression of ENKUR results in decreased cell migration as revealed by cell wound healing assay. Images were taken at 0 and 24 h. (C) Overexpression of ENKUR inhibits cell growth as determined by colony formation assay. Both the number of colonies and colony formation efficiency were significantly lower in A549-ENKUR cells than in the control cells. (D) Overexpression of ENKUR results in decreased migration and invasion through extracellular matrix as revealed by the Transwell migration assay. Representative photographs (left) and quantification (right) are shown. The number of cells migrated through the extracellular matrix (ECM) after 24 h was counted in five randomly selected (×200) microscopic fields. Each error bar represents the mean ± S.D. of three replicate samples. * *p* < 0.05.

A stable ENKUR-overexpressed LAD cell line, A549-ENKUR was constructed to investigate the role of ENKUR in regulating the biological behavior of LAD cells. A549 cells transduced with empty lentiviral vectors were used as negative control. A549-ENKUR cells showed significantly higher ENKUR expression compared with the control cells, as revealed by qRT-PCR and western blot analyses (*p<*0.05, Figure 3). Overexpression of ENKUR in LAD cells strongly inhibited cell proliferation and migration. In MTT assay and colony formation assay, the A549-ENKUR cells showed significantly lower growth rate compared with the control cells (*p <* 0.05; Figure 4A and C). Meanwhile, slower wound closure and reduced migratory rate were observed in A549-ENKUR cells, as revealed by wound healing assay and Transwell migration assay (*p<*0.05; Figure 4B and D).

### Silencing of ENKUR promotes the proliferation, migration, invasion, and tumourigenesis of human LAD cells

**Figure 5:**
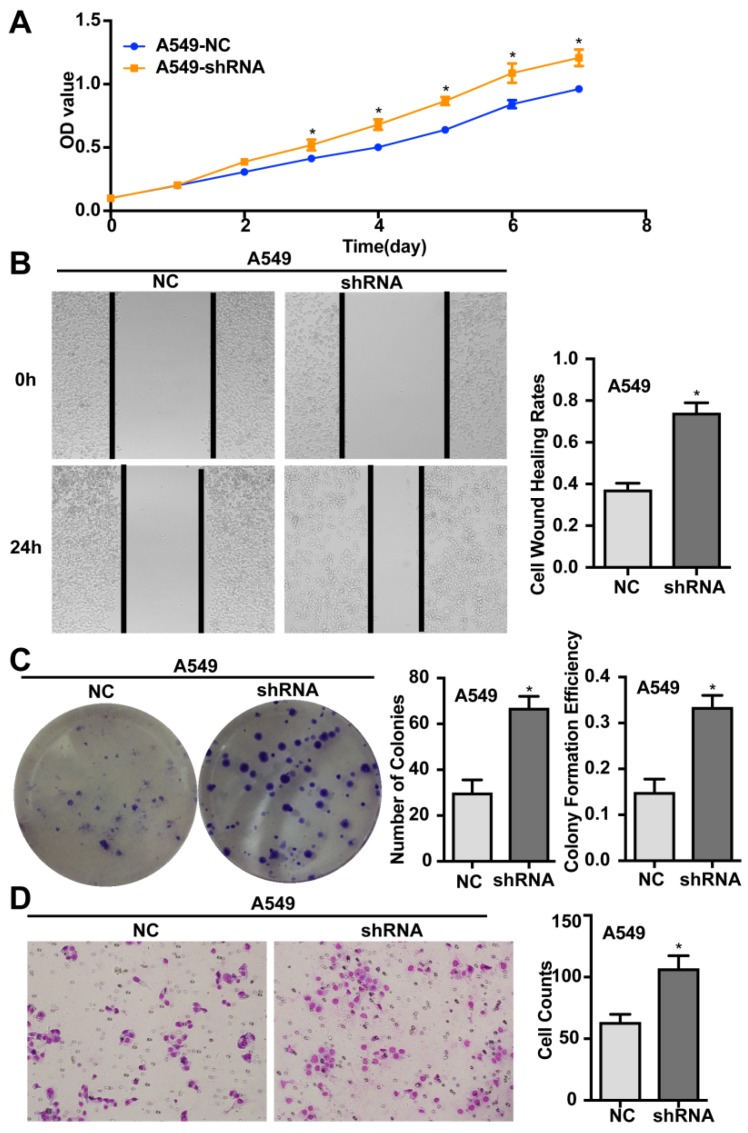
** Silencing of ENKUR promotes proliferation, migration, and invasion of LAD cells.** (A) ENKUR-silenced cells A549-shRNA exhibit enhanced cell proliferation compared with the control groups. (B) Silencing of ENKUR provokes stronger cell migration of LAD cells. Images were taken at 0 and 24 h. (F) Silencing of ENKUR promotes cell growth as determined by the colony formation assay. Both the number of colonies and colony formation efficiency were significantly higher in A549-shRNA cells than in the control cells. (H) Silencing of ENKUR results in enhanced migration and invasion through extracellular matrix as revealed by the Transwell migration assay. Representative photographs (left) and quantification (right) are shown. The number of cells migrated through the extracellular matrix (ECM) after 24 h was counted in five randomly selected (×200) microscopic fields. Each error bar represents the mean ± S.D. of three replicate samples. * *p* < 0.05.

**Figure 6:**
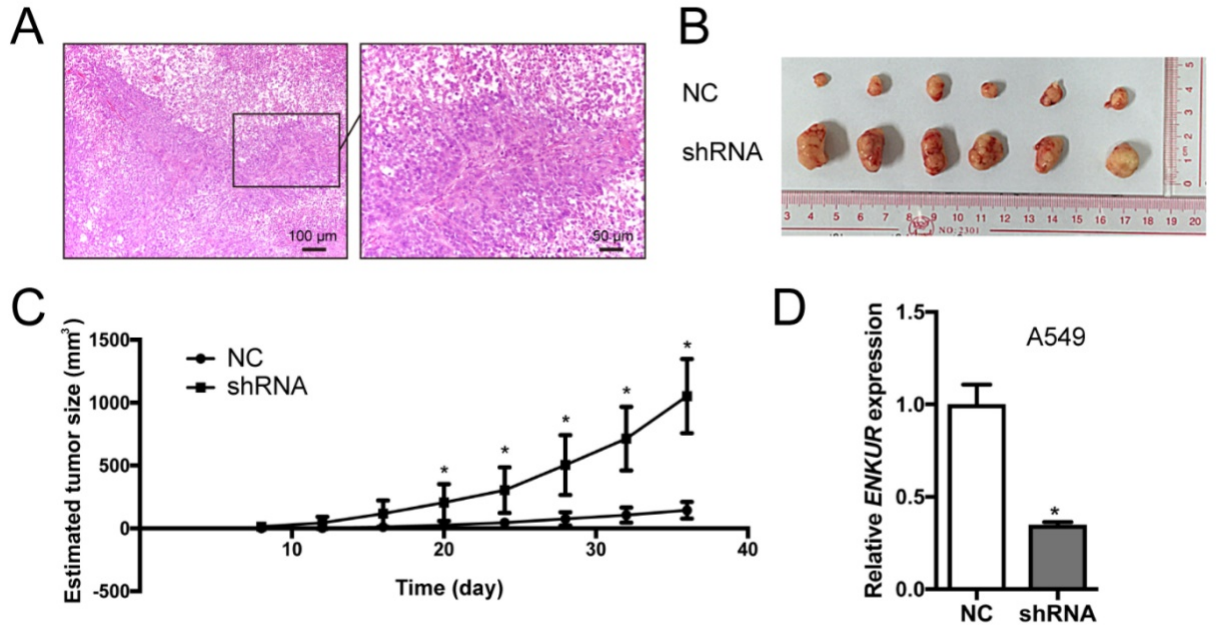
** Silencing of ENKUR promotes tumor growth in nude mice model.** (A) Hematoxylin-Eosin (H&E) of a xenograft tumor. (B) Gross observation of xenograft tumor formed of the ENKUR-silenced A549 cells (shRNA) and the control cells (NC). (C) Statistical chart of tumor size. (D) qRT-PCR analysis of *ENKUR* expression in xenograft tumors. Each error bar represents the mean ± S.D. of three replicate samples. * *p* < 0.05.

Silencing of endogenous ENKUR expression in LAD cells A549 was achieved using a lentiviral vector carrying a specific shRNA. The silencing efficiency was confirmed by qRT-PCR and western blot analyses (Figure 3). Silencing of ENKUR in LAD cell lines provoked accelerated cell growth and migration. In MTT assay and colony formation assay, the A549-shRNA cells demonstrated a significant growth advantage over the control cells (*p <* 0.05; Figure 5A and C). Meanwhile, more rapid wound closure and higher migratory rate were observed in A549-shRNA cells, as revealed by wound healing assay and Transwell migration assay (*p<*0.05; Figure 5B and D). Consistent with this effect, *in vivo* xenograft assay showed that tumors formed of A549-shRNA cells grew more rapidly than tumors generated from the control cells (*p*<0.05; Figure 6). Palpable tumors were detected on day 8 after injection.

### ENKUR silencing alters cell phenotype

**Figure 7:**
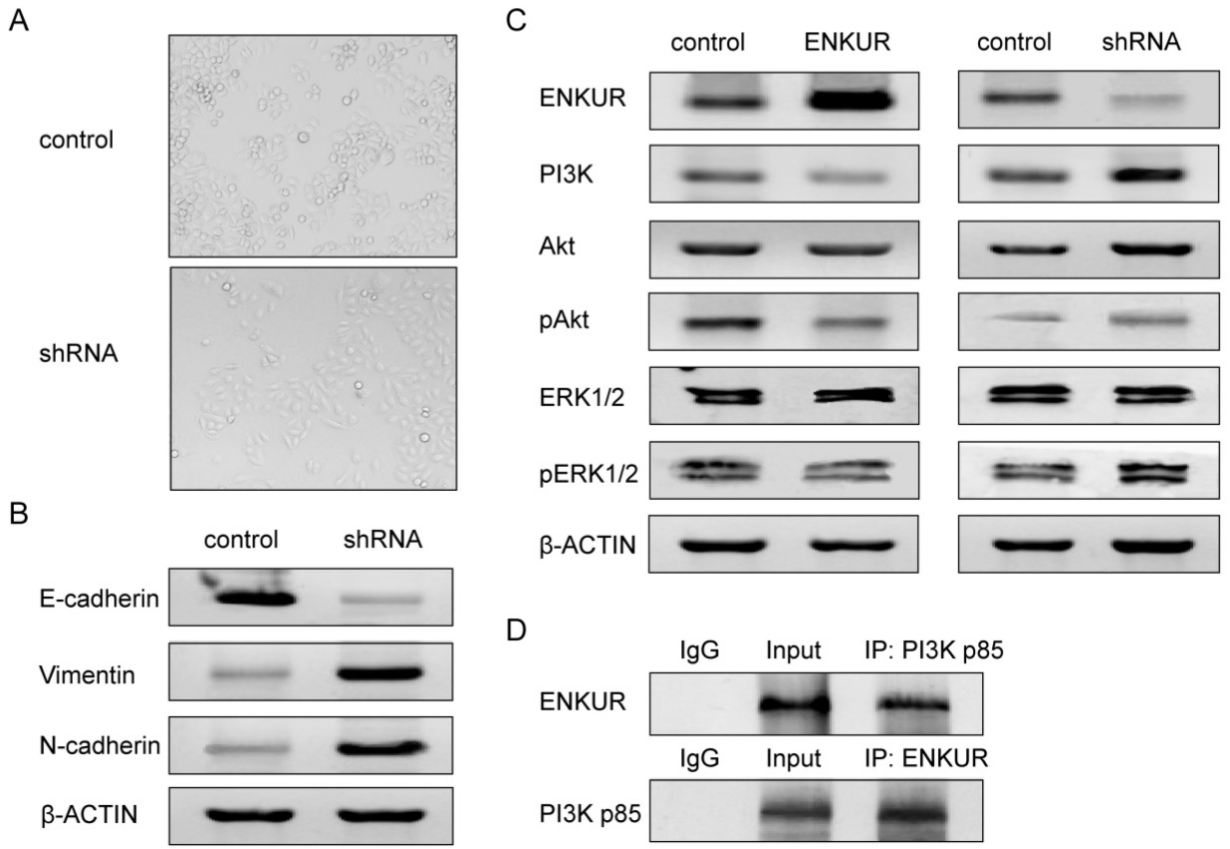
** ENKUR is associated with EMT and the PI3K/Akt and MAPK/ERK signaling pathways in LAD cells.** (A) Silencing of ENKUR in LAD cells A549 caused phenotypic switch from non-invasive epithelial cells to mesenchymal, spindle cells. (B) Silencing of ENKUR in LAD cells A549 induces hallmarks of the EMT, including loss of E-cadherin and accumulation of N-cadherin and vimentin. (C) Increased PI3K expression and phosphorylation levels of Akt and ERK1/2 were detected in ENKUR-silenced LAD cells, while inhibited activities of PI3K/Akt and MAPK/ERK pathways were observed in ENKUR-overexpressed LAD cells. (D) ENKUR co-immunoprecipitated with PI3K p85 in LAD cells. Lysates from A549 cells were immunoprecipitated with ENKUR antibody or control IgG and detected with PI3K p85 antibody on a western blot, then immunoprecipitated with PI3K p85 antibody or control IgG and detected with ENKUR antibody on a western blot.

Comparison between the A549-shRNA cells and control cells showed that silencing of ENKUR caused switch from non-invasive epithelial cells to mesenchymal, spindle cells (Figure 7A). This phenotypic change suggested induction of epithelial-mesenchymal transition (EMT). We therefore further investigated the expression of markers associated with EMT. Western blot analyses showed that silencing of ENKUR significantly decreased E-cadherin expression and increased N-cadherin and vimentin expression (Figure 7B).

### ENKUR is involved in the PI3K/Akt and MAPK/ERK signaling pathways

We next investigated the possible pathways that ENKUR may be involved in. Increased PI3K expression was detected in ENKUR-silenced cells, while decreased PI3K expression was detected in ENKUR-overexpressed cells (Figure 7C). Co-immunoprecipitation analysis revealed protein-protein interaction between ENKUR and PI3K p85 (Figure 7D). Meanwhile, overexpression of ENKUR led to decreased phosphorylation levels of Akt and ERK1/2. In contrast, silencing of ENKUR resulted in evidently increased phosphorylation levels of Akt and ERK1/2 as compared with the control group (Figure 7C).

## Discussion

Carcinogenesis is a multistep process comprising a series of biological events. Among the many signaling pathways that orchestrate cell activities including proliferation, survival, differentiation, and motility, the PI3K/Akt and MAPK/ERK pathways have long been implicated in the pathogenesis of human carcinoma 14. Constitutive activation of PI3K/Akt and MAPK/ERK pathways resulting from molecular alteration of the intracellular phosphor-signaling cascade components is frequently observed in human cancer 15-17. Accumulating evidences have suggested that both the PI3K/Akt and MAPK/ERK pathways represent promising therapeutic targets. Several inhibitors of these pathways are currently undergoing evaluation in preclinical studies of lung cancer 18. However, due to the complex and dynamic genetic network and various regulators associated with PI3K/Akt and MAPK/ERK signaling, a more comprehensive molecular portrait of these pathways is a prerequisite to thoroughly understand the molecular mechanisms behind cancer progression and to precisely predict the risk of metastatic recurrence 14, 19.

Located at chromosomal band 10p12.1, the *ENKUR* (*Enkurin*) gene encodes a CaM-binding protein that directly interacts with the p85 regulatory subunit of PI3K^8^. Here we analyzed the expression level of *ENKUR* in clinical samples of LAD using the UALCAN web server based on TCGA database and the Oncomine platform. Compared with normal tissues, *ENKUR* was significantly down-regulated by approximately 50% in LAD as well as in colon adenocarcinoma, kidney chromophobe, lung squamous cell carcinoma, and thyroid carcinoma (*p*<0.05; Figure 1). Consistent with the expression profiles of *ENKUR* in public databases, our western blot and qRT-PCR analyses showed down-regulated *ENKUR* mRNA expression and ENKUR protein level in five LAD cell lines as compared with the normal bronchial epithelial cell line 16HBE (Figure 2). Moreover, silencing of ENKUR increased cell proliferative, migratory, and invasive properties *in vitro* and promoted tumor growth *in vivo* (Figures 4, 5, 6). We believe these data support a potential role of ENKUR in controlling cellular behavior and tumorigenesis of LAD cells.

Epithelial-mesenchymal transition (EMT) is a key process in cancer metastasis. Epithelial cancer cells undergoing EMT tend to lose their cell-cell adhesive properties and gain migratory and invasive potential, resulting in local tissue invasion and metastasize 20, 21. Changes in the expression of EMT markers were found to predict poor prognosis in LAD 22. In this study, transformation from epithelial to mesenchymal phenotype accompanied by reduced expression of E-cadherin and increased expression of N-cadherin and vimentin were observed in ENKUR-silenced A549 cells (Figure 7A and B). Since ENKUR was found to interact directly with the p85 subunit of PI3K, which plays an oncogenic role in lung cancer by regulating cancer stem cell growth, survival, and metabolism 23-25, we also investigated changes in the activities of PI3K/Akt signaling pathway and its closely-related MAPK/ERK signaling pathway in ENKUR-overexpressed and -silenced cells. Overexpression of ENKUR caused significantly reduced PI3K expression and decreased phosphorylation of Akt and ERK1/2 as compared with the control group. By contrast, silencing of ENKUR led to increased activities of PI3K/Akt and MAPK/ERK signaling (Figure 7C). Meanwhile, co-immunoprecipitation analysis revealed protein-protein interaction between ENKUR and PI3K (Figure 7D). This confirms the previous finding that ENKUR binds to the p85 subunit of PI3K 7. It is noteworthy that activation of the MAPK/ERK and PI3K/Akt pathways is an important step required for EMT process induced by various tumor-related factors including TGF-β, TACC3, EGF, BMP7, ZNF143, and CXCR4 26-31. Combined with our results, we postulate that ENKUR may interfere with EMT in LAD cells by modulating the activities of PI3K/Akt and MAPK/ERK signaling. Similarly, several cell signaling regulators and tumor suppressor microRNAs also function through PI3K/Akt and MAPK/ERK signaling pathways, reversing the EMT process and ultimately suppressing oncogenic transformation and cancer progression 32, 33. Since previous studies have revealed a cross-talk between the PI3K/Akt and MAPK/ERK pathways, blocking a single pathway will probably give rise to compensatory activation of the other pathway. Hence, inhibitors of both the PI3K/Akt and MAPK/ERK pathways may result in a more significant antitumor effect 14. This implicates the potential of ENKUR in clinical use in the future and makes ENKUR an interesting target for further investigation.

In conclusion, our findings provide evidence for a novel anti-tumor role of ENKUR in LAD. We propose that ENKUR is down-regulated in LAD and silencing of ENKUR may be responsible for malignant transformation in LAD cells, leading to phenotypic change and enhanced proliferation, migration, and invasion. In addition, ENKUR can directly interact with PI3K and is possibly involved in the PI3K/Akt and MAPK/ERK signaling pathways. Further molecular studies of the genetic basis underlying the down-regulation of *ENKUR* in LAD and identification of co-regulators of ENKUR will help unravel the intrinsic mechanism of ENKUR-mediated carcinostatic effect and develop new gene therapies for patients with LAD.

## Figures and Tables

**Table 1:** Primer sequences used for qRT-PCR analysis

Gene	Primer	Oligonucleotide	Product Size
*ENKUR*	Forward (5'-3')	TATGGCCGGGGAGAAATAGG	473 bp
	Reverse (3'-5')	GTTTTTCGGACGACACGGTA	
*β-ACTIN*	Forward (5'-3')	AGCGGGAAATCGTGCGTG	309 bp
	Reverse (3'-5')	CAGGGTACATGGTGGTGCC	
